# Effect of insulin and cinnamon extract on spatial memory and gene expression of GLUT1, 3, and 4 in streptozotocin-induced Alzheimer’s model in rats

**DOI:** 10.22038/IJBMS.2023.68568.14957

**Published:** 2023

**Authors:** Elham Sajadi, Javad Sajedianfard, Saeid Hosseinzadeh, Mahnaz Taherianfard

**Affiliations:** 1 Department of Basic Sciences, School of Veterinary Medicine, Shiraz University, Shiraz, Iran; 2 Department of Food Hygiene and Public Health, School of Veterinary Medicine, Shiraz University, Shiraz, Iran

**Keywords:** Alzheimer’s disease, Cinnamomum cassia, GLUT1, GLUT3, GLUT4, Insulin

## Abstract

**Objective(s):**

Since diminished hippocampal insulin signaling leads to memory impairment, insulin resistance and hyperinsulinemia are probably associated with Alzheimer’s disease (AD). The effect of intracerebroventricular injection of insulin (Ins) and oral cinnamon extract (Cinn) on glucose transporter (GLUT) 1, 3, and 4 gene expressions in the hippocampus and spatial memory in a streptozotocin (STZ)-induced AD rat model was investigated in the present study.

**Materials and Methods:**

Fifty-six adult male Sprague-Dawley rats (280±20 g) were allocated into eight distinct groups (n=7) of five controls (negative, Ins, Cinn, Ins+Cinn, and STZs) and three treatments (STZ+ Ins, STZ+ Cinn, and STZ+ Ins + Cinn). Single dose STZ 4 mg/kg (icv), Cinn at a dose of 200 mg/ kg (orally for 14 days), and Ins 5 mIU/5 µl (icv for 14 days) were administered in the defined groups. To evaluate the behavioral performance the animals were subjected to the Morris Water Maze (MWM) test. The level of mRNA expression of GLUTs was evaluated by the Real time-PCR method.

**Results:**

In the STZ+Cinn+Ins group, the performance of animals in the MWM test was improved and the over-expression of GLUTs genes in hippocampal tissue was observed. The results of Ins and Cinn synergist treatment groups revealed improvement in the behavioral tests and gene expression compared with Ins and Cinn treatment groups (*P*<0.001).

**Conclusion:**

Administration of Ins and Cinn has a positive effect on the function of the AD rat model. To clarify the effect of Ins and Cinn extract on the GLUTs investigated in this study, it is essential to evaluate their influence on the protein levels.

## Introduction

Besides types 1(T1DM) and 2 (T2DM) diabetes mellitus, another form of the disease, namely type 3 diabetes mellitus (T3DM), has recently been identified. T3DM is attributed to insulin resistance in the brain and is accompanied by Alzheimer’s disease (AD) (1). Insulin (Ins), a modulator of β-amyloid (Aβ) levels, stimulates the release of Aβ from the intracellular to extracellular compartments (2). Degradation of Aβ is through the insulin-degrading enzyme (IDE) by the extracellular consequence of insulin (3). AD, the fifth key factor of death in people above 65 years of age (1), is associated with the deficiency of neurotransmitters, destruction of neurons, synaptic dysfunction, extracellular accumulation of Aβ, tau hyper-phosphorylation, and intracellular formation of neurofibrillary tangles (NFT) (4).

D-glucose, the principal origin of the brain’s energy, requires special transporters called glucose transporters (GLUTs) to move across the blood-brain barrier (BBB) and deliver absorbed glucose to astrocytes and neurons. GLUTs and astrocytes contribute to the cellular glucose balance of the brain (5). So far, 10 GLUT proteins have been recognized in the nervous system, and GLUT1 and 3 play pivotal roles in brain glucose uptake. GLUT4 is present in some neurons in the brain, hippocampus, and cerebellar neurons (6). One of the models of Alzheimer’s induction that reduced cerebral glucose uptake, causing multiple molecular, pathological, and behavioral effects of AD, is an intracerebroventricular injection of Streptozotocin (STZ-icv) (7). One of the side effects of STZ-icv is an increase in Aβ deposition in the hippocampus due to a decrease in IDE levels (8). Alternatively, STZ-icv causes disturbances in the brain energy metabolism, the glucose transport system derangement, disorders of metabolic pathways under the control of Ins signaling, inhibition of ATP and acetyl CoA synthesis, and decreases hippocampal choline acetyltransferase activity. All of which cause cognitive impairment in the animal model (9, 10).

Various studies have been currently focused on the influences of natural products and bioactive compounds on neurodegenerative diseases (11). *Cinnamomum*, a genus of evergreen aromatic trees, belongs to the Lauraceae family which increases the expression of glucose transporter proteins and Ins signaling (12) and has preventive and therapeutic potential for Parkinson’s and Alzheimer’s diseases (13). Cinnamon (Cinn) contains cinnamaldehyde, eugenol, cinnamic acid, tannin, catechin, proanthocyanidin, monoterpenes, cespine terpenes, and coumarin. Cinn has been shown to modulate glycogenesis and gluconeogenesis, reduce glucose and blood glucose tolerance in diabetic rats, increase Ins secretion in the islets of Langerhans (14), and due to the presence of vinyl parts has antioxidant activity leading to memory enhancement (15). The previous finding on the advantage of Cinn in AD suggested that the trans-cinnamaldehyde improves AD pathology by reducing β-secretase levels, the first rate-limiting enzyme in Aβ production, through activation of the silent information regulator 1, peroxisome proliferator-activated receptor γ coactivator 1α (PGC)-1α, and PPARγ pathway (16). Also, the Cinn treatment in a non-transgenic rat model of AD could improve Ins sensitivity, increase phosphorylated glycogen synthase kinase-3β (pGSK3β), inhibit cholinesterase activity, and improve learning (17).

The simultaneous effect of Cinn and Ins on spatial memory by evaluating their impact on the expression of hippocampal GLUTs genes has not as yet been studied. In the present study, the effects of orally administered *Cinnamomum cassia *extract alone or in combination with the icv injection of Ins on the water maze test results and the level of mRNA GLUTs expression in an STZ-induced rat model of AD are investigated. 

## Materials and Methods

***Animals groups and ethical approval***

Fifty-six adult male Sprague-Dawley rats (280±20 g) were purchased from the Animal Center of Shiraz Medical University and maintained under the standard conditions of 22±2 °C , 12 hr light/dark cycle, the animals had free access to food and water for one week before commencing the experiments. 

Procedures adopted in this study followed Shiraz University’s ethical guidelines for use of animals in the experimental studies (97GCU2M1293).

***Preparation of cinnamon extract***

Cinnamon-tree trunk’s bark was purchased from herbal drug stores in Shiraz (Iran); It was identified as *C. cassia* by the National Botanical Research Institute, Shiraz, Iran. The Cinn extract was prepared as follows: 500 g of Cinn powder was mixed with 2 L of 70% ethanol; shaken for 48 hr; filtered with Whatman filter paper; evaporated to remove the ethanol; lyophilized and stored at 4 °C.

***Experimental protocols***

*Groups*

Rats were indiscriminately separated into eight groups (n=8) including five groups of control and three experimental groups as follows: negative control (aCSF) (10 µl); insulin control (Ins); cinnamon control (Cinn); insulin and Cinn control (Cinn+Ins); STZ; treatment1 (STZ+Ins); treatment2 (STZ+Cinn); treatment3 (STZ+Ins+Cinn). aCSF was applied in all control groups.

**Figure 1:**
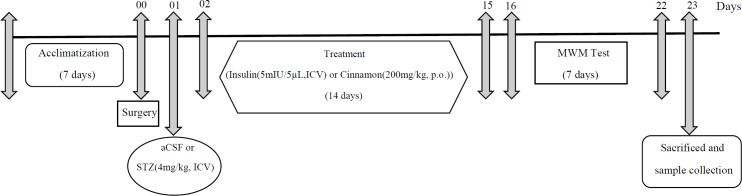
Experimental protocols for evaluation of insulin and cinnamon in streptozotocin-induced Alzheimer’s model in rats

Cinn extract (orally by gavage) and Ins (icv) at doses of 200 mg/ kg and 5 mIU/5 µl for 14 days, respectively (18, 19); and STZ (icv) at a single dose of 4 mg/kg (8) were administered (Figure 1).

*Induction of Alzheimer’s in the animal model*

Animals were anesthetized with Ketamine (100 mg/kg, IP) (Alfasan, Netherlands) and Xylazine (8 mg/kg) (Alfasan, Netherlands), then set down in a stereotaxic device (Stoleting, USA) with the incisor bar set 3.5 mm below the inter-aural line. A cannula was implanted in the lateral ventricle (AP= -0.8, LR=1.5 mm, D=3.6 mm) (Paxinos & Watson, 2010). In the induction models, STZ (4 mg/kg) was dissolved in aCSF and injected icv by 10 µl Hamilton micro-syringe (speed of injection 1 µl/min) (8). 

*Morris Water Maze (MWM)*

Two weeks after surgery, the MWM test was performed. A circular black pool (170 cm diameter × 60 cm high), filled with water (25±2 °C ; depth of 40 cm), was divided into four special visual clues including North East (NE), North West (NW), South East (SE), and South West (SW). A black platform (10×10 cm) was placed in the SE quadrant and submerged 1.5 cm beneath the water in the hidden platform test. All tests were done from 8:00 AM to 2:00 PM. A digital camera was mounted above the pool to capture images per second in each trial and transmit them to a computer smart software (Neurovision, Iran).

During the first three days, the rats were examined in the visible platform test and had to find the platform within 90 sec. If they failed, the operator would have to guide them to the platform gently and allow them to stay there for 60 sec. In the later three days, the ratsʼ reference memory was examined in the hidden platform test, swam in the pool to find the submerged platform within 60 sec. For both visible and hidden platform tests, each animal was contributed in four assessment groups per day for three following days, albeit with dissimilar starting points; the escape latency time and swimming speed were recorded in these steps. On the seventh day, a probe test was performed; the platform was taken out and each rat was permitted to swim for 60 sec. The percentage of time spent in the target quadrant was recorded as an index of the probe test.

***Preparations of tissue samples***

After the behavioral tests, the animals were euthanized by CO_2_, the brains were detached for isolation of the hippocampus. The hippocampus was immediately transferred in RNase-free microtubes. Then, 0.3 g of the tissue was homogenized with RNX-Plus solution.

***Expression of genes***

*RNA isolation and cDNA synthesis*

The left hippocampus RNAs were extracted with RNX-Plus solution as was described by the manufacturer (CinnaGen Co, Iran). RNA quality was tested by a Nanodrop spectrophotometer (Thermo Scientific, USA) and only those in frames of A260/A280>1.80 were selected. In the synthesis of complementary DNAs (cDNA), 3 µg of total RNA (Yekta Tajhiz Azma cot N YT4500) were mixed with 1 µl of oligo dT primer and delivered to DEPC-treated water to a volume of 13.4 μl. A cDNA synthesis mix (6.5 µg) was added to each tube and incubated for 60 min at 42 °C. The terminal response was induced by heating (70 °C for 5 min). 

*Real-time PCR assay*

Real-time PCR amplification was accomplished using SYBR Green I PCR Master Mix 2x kit (Sinuhe, Iran) in Light Cycler 480® (Roche, Germany). The PCR reaction was done in triplicate as follows thermocycling conditions: 95 °C for 10 min, followed by 40 cycles of 95 °C for 20 sec, 58 °C for 30 sec, 72 °C for 30 sec, and the final step 72 °C for 5 min. The average expression of GAPDH as an internal reference gene to normalize the input cDNA was applied. The method for calculating the fold change was 2^-ΔΔCt^. Table 1 showed the list of primers used in this study.

***Statistical analysis***

SPSS software version 23 (SPSS, Inc., Chicago, IL, USA) was used to analyze the data. In addition, the Repeated Measures ANOVA was employed to analyze the data relating to the visible and hidden platforms and the Bonferroni supplementary test. Other data were analyzed by one-way analysis of variance and Duncan’s supplementary test. The Real-time PCR results were analyzed by Light cycler 96 version 1.1. All data were described as mean ± standard deviation (Mean ± SD) and a significance level of *P*<0.05 was considered to compare the data.

## Results

***Behavioral results***

*Escape latencies visible platform training*

**Figure 2:**
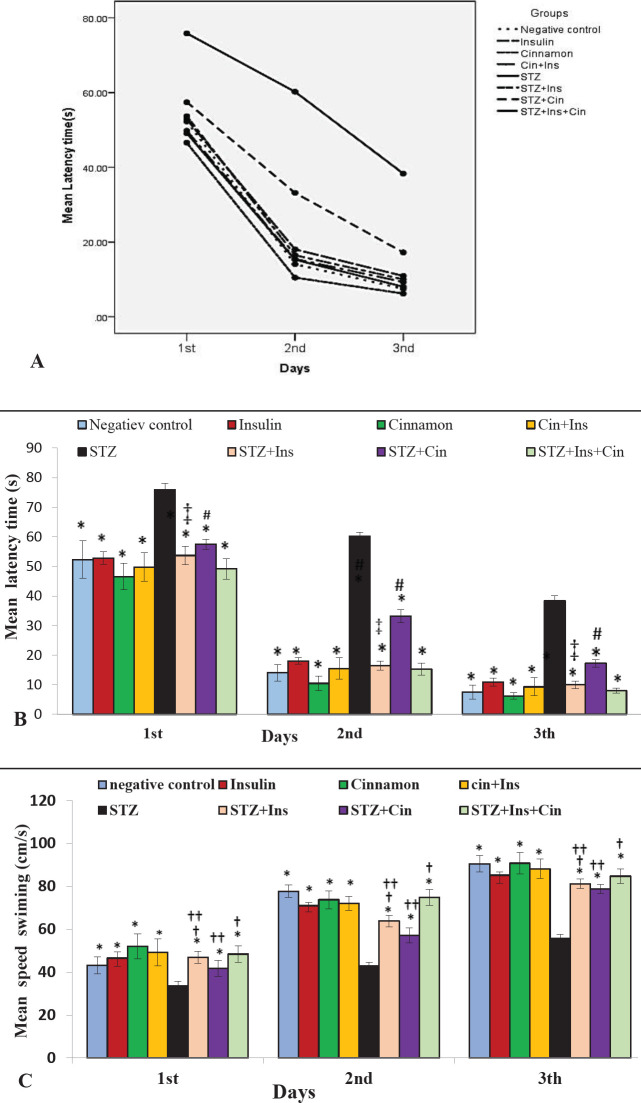
Mean escape latency time and mean swimming speed to clarify the visible platform in a water maze. A) Learning patterns of the animals in different groups. The decreasing trend of the time elapsed to reach the target platform can be seen in different groups from the first to the third day; B) Mean escape latency to the visible platform during days 1–3 of training; C) Mean swimming speed to find the visible platform during days 1–3 of training; Eight groups (n=7) were tested: Negative control; Insulin control (Ins); Cinnamon control (Cinn); Cinnamon+Insulin control (Cinn+Ins); STZ; STZ+ Insulin (STZ+Ins); STZ+Cinnamon (STZ+Cinn); STZ+ Insulin+ Cinnamon (STZ+Ins+Cinn); (cinnamon extract at a dose of 200 mg/kg (orally by gavage); STZ 4 mg/kg (icv); Insulin 5 mIU/5 µl (icv)). Error bar indicates standard deviation.

Statistical analysis of repeated measures revealed that there was a significant difference between the groups in connection with escape latency time during the visible platform training (*P*<0.001 and (F (7,48) = 216.87, *P*<0.001, partial Eta squared = 0.97) (Figure 2). 

The latency variable for finding the obvious platform was significantly different between the groups. The time to achieve the target platform in the STZ + Cinn group was longer between the treatment groups, and the difference between the result of this group and other experimental groups was significant (*P*<0.001). In the treatment groups, the STZ +Ins+Cinn group reached the target platform in a shorter time. Also, the STZ + Ins + Cinn group reached the platform in a shorter time than STZ + Cinn group revealing a significant difference between them (*P*<0.001). Compared with the STZ group, all groups reached the platform in a shorter time (*P*<0.001). In addition, in comparison between the days, the Bonferroni test showed a statistically significant difference between the days, and from the first to the third day, the delay time to reach the target platform has a decreasing trend (Figure 2).

*Swimming speed to find the visible platform*

Statistical analysis of repeated measures displayed that there was a significant difference between the groups regarding swimming speed during the visible platform training (F = 89.97, *P*<0.001, partial Eta squared = 0.93 ) and there was a significant difference between all groups and the STZ group (*P*<0.001) (Figure 2). Between the treatment groups, the performance of the STZ + Cinn group was weaker. That is, the speed of swimming to reach the platform was higher in this group and there was a significant difference between all treatment groups (*P*<0.001). The results of the STZ + Ins + Cinn group were better than other treatment groups; the swimming speed to reach the platform was slower in this group and there was a significant difference compared with other treatment groups (*P*<0.001). In comparison between the control and treatment groups, the speed of swimming in the STZ + Cinn and STZ + Ins groups was higher compared with the Cinn and Ins + Cinn control groups (*P*<0.001). In addition, the comparison between the days, the Bonferroni test showed that there was a significant difference between the days in terms of swimming speed; from the first to the third day, the swimming speed increased to reach the target platform (*P*<0.001). 

*Escape latencies hidden platform training*

**Figure 3:**
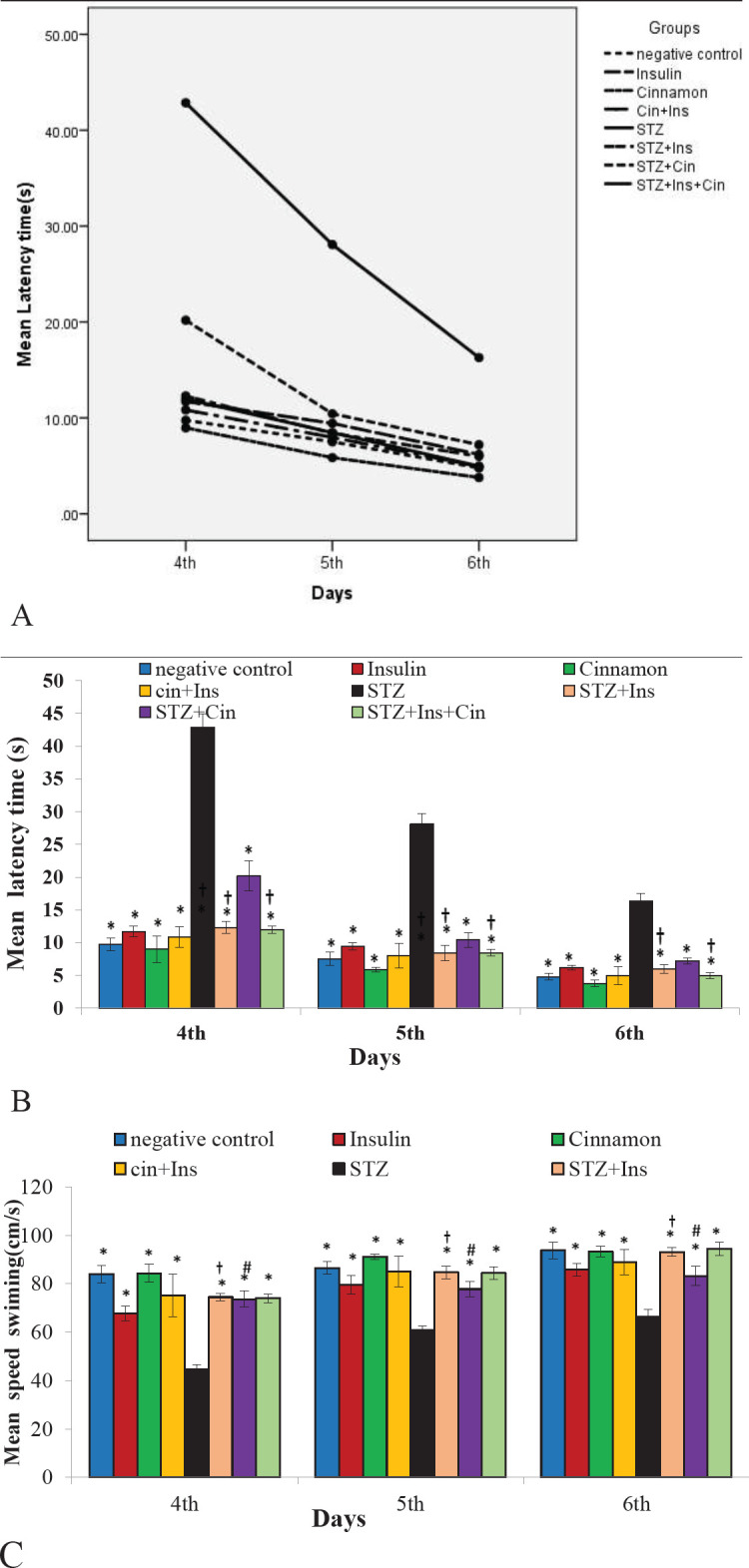
Mean escape latency time and mean swimming speed to find the hidden platform in a water maze. A) Learning outlines of the animals in various groups. The decreasing trend of the time elapsed to reach the target platform can be seen in different groups from the 4^th^ to the 6^th^ day; B) The mean escape latency to the hidden platform during days 4–6 of training; C) The mean swimming speed to find the hidden platform during days 4-6 of training; Eight groups (n=7) were tested: Negative control; Insulin control (Ins); Cinnamon control (Cinn); Cinnamon+Insulin control (Cinn+Ins); STZ; STZ+ Insulin (STZ+Ins); STZ+Cinnamon (STZ+Cinn); STZ+ Insulin+ Cinnamon (STZ+Ins+Cinn); (Cinnamon extract at a dose of 200 mg/ kg (orally by gavage); STZ 4 mg/kg (icv); Insulin 5 mIU/5 µl (icv)). Standard deviation is indicated by the error bar

Statistical analysis of repeated measures displayed a significant difference between the groups concerning the latency time during the hidden platform training (F (7,48) = 525.35, *P*<0.001, partial Eta squared = 0.99) (Figure 3). In the treatment groups, STZ + Ins and STZ + Ins + Cinn groups, the delay time to find the hidden platform was shorter compared with the STZ + Cinn group (*P*<0.001). All groups had a statistically significant difference from the STZ group and the latency to find a hidden platform in them was shorter compared with the STZ group (*P*<0.001).

In comparison between days, the Bonferroni test exhibited a statistically significant difference between days regarding the latency to find the hidden platform (*P*<0.001) (Figure 3).

*Swimming speed to find the hidden platform*

Statistical analysis of repeated measures indicated a significant difference between the groups in terms of swimming speed during the hidden platform training (F 78.68, *P*<0.001, partial Eta squared = 0.92), and swimming speed to find the platform in all groups was higher compared with the STZ group (*P*<0.001) (Figure 3). Between the treatment groups, the swimming speed in STZ + Ins and STZ + Ins + Cinn groups was higher than the others and there was a significant difference with STZ + Cinn group. In addition, the Bonferroni test showed that the swimming speed during the test days had a significant difference and from the first to the third day, the swimming speed was faster.

*Probe test*

**Figure 4:**
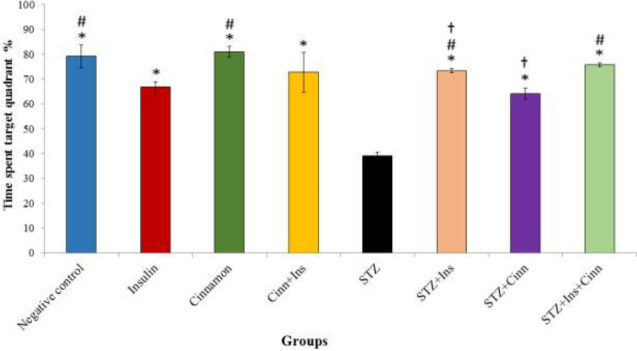
Percentage of time spent in the target quadrant in a water maze probe test. Eight groups (n=7) were tested: Negative control; Insulin control (Ins); Cinnamon control (Cinn); Cinnamon+Insulin control (Cinn+Ins); STZ; STZ+ Insulin (STZ+Ins); STZ+Cinnamon (STZ+Cinn); STZ+ Insulin+ Cinnamon (STZ+Ins+Cinn); (cinnamon extract at a dose of 200 mg/ kg (orally by gavage); STZ 4 mg/kg (icv); Insulin 5 mIU/5 µl (icv)). Error bar indicates standard deviation

To do this step, the platform in the target quadrant was removed; then, the percentage of time spent in the target quadrant was recorded. On the 7^th^ day of the experiment, the mean spending time was significantly different (*P*<0.001) between the studied groups (Figure 4). The percent of time spent in the target quadrant in all groups was more than the STZ group (*P*<0.001). Also, a statistically significant difference was observed between all experimental groups; the highest time spent in the target quadrant was revealed in the STZ+ Ins+ Cinn treatment group (*P*<0.001). Comparing the STZ+Ins treatment group and the insulin control, the time in the former group was longer than that in the latter group; however, such a result was not observed in the comparison between the STZ+Cinn treatment and the Cinn control groups. Interestingly, Ins alone hurt the performance of animals in the probe test; however, cinn positively affected spatial memory in the probe test.

***Gene expression in the hippocampus***

*Expression of GLUT1 mRNA in the hippocampus*

**Figure 5:**
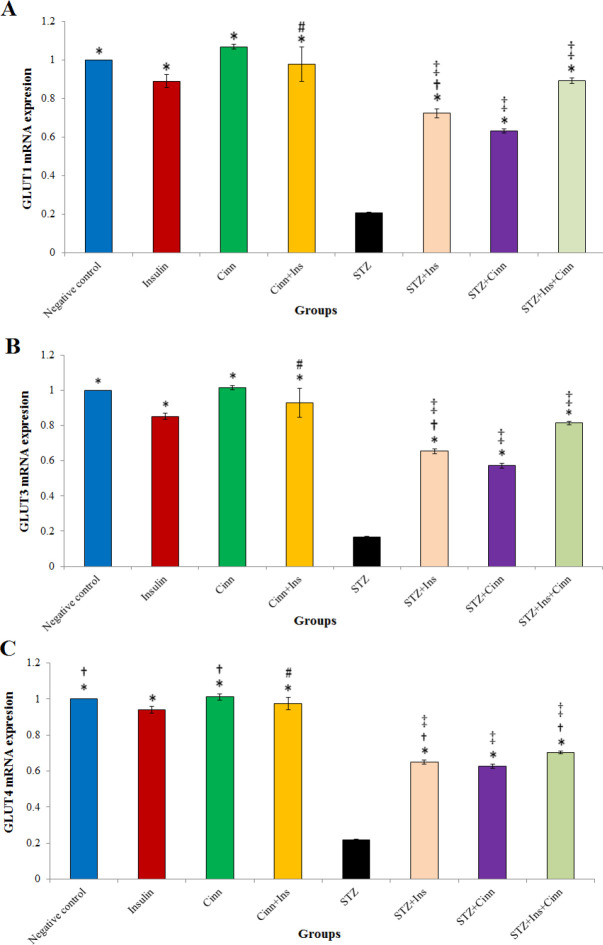
Mean of mRNA expression of (A) GLUT1, (B) GLUT3, and (C) GLUT4 levels of hippocampus tissue in different groups. Eight groups (n=7) were tested: Negative control; Insulin control (Ins); Cinnamon control (Cinn); Cinnamon+Insulin control (Cinn+Ins); STZ; STZ+ Insulin (STZ+Ins); STZ+Cinnamon (STZ+Cinn); STZ+ Insulin+ Cinnamon (STZ+Ins+Cinn); (cinnamon extract at a dose of 200 mg/ kg (orally by gavage); STZ 4 mg/kg (icv); Insulin 5 mIU/5 µl (icv)). Error bar indicates standard deviation

The expression of the GLUT1 gene in the hippocampus was significantly higher in all groups compared with the STZ group (*P*<0.001). The gene expression in the STZ+Cinn+Ins group was higher than other treatment groups (STZ+Cinn and STZ+ Ins) (Figure 5). The highest gene expression was observed in the negative control group, cinnamon control, Cinn+Ins, and Ins control; the lowest gene expression was observed in the STZ group and STZ+Cinn treatment group. There was a significantly (*P*<0.001) higher gene expression in the Ins control group compared with the STZ+Ins group. This result was also true in the Cinn control group and STZ+Cinn (*P*<0.001). No statistically significant difference was found between the Cinn+Ins group and STZ+Cinn+Ins.

*GLUT3 mRNA expression in the hippocampus*

The expression of the GLUT3 gene in the hippocampus was significantly higher in all groups compared with the STZ group (*P*<0.001) (Figure 5). Between the treatment groups, the STZ+Ins+Cinn group had a significantly (*P*<0.001) higher gene expression than the other two experimental groups (*P*<0.001). The highest levels of GLUT3 mRNA expression in the hippocampus were observed in four groups: Cinn control, negative control, Cinn+Ins control, and treatment STZ+Cinn+Ins, respectively. The lowest gene expression was observed in three groups of STZ, treatment STZ+Cinn, and STZ+Ins, respectively.

*GLUT4 mRNA expression in the hippocampus*

The expression of GLUT4 mRNA in hippocampal tissue was significantly reduced in the STZ group compared with other experimental groups (Figure 5). Between treatment groups, GLUT4 mRNA expression was higher in the STZ+Ins+Cinn group in comparison with the STZ+Ins and STZ+Cinn groups (*P*<0.001). The highest GLUT4 mRNA expression was recorded in the Cinn control, negative control, Cinn+Ins control, and Ins control groups. The lowest gene expression was in the STZ, STZ+Cinn, and STZ+Ins groups. The overexpression of gene in the Ins control group compared with the STZ+Ins (*P*<0.001); in the Cinn control, more than STZ+Cinn (*P*<0.001); and in the Cinn+Ins, more than STZ+Cinn+Ins (*P*<0.001).

## Discussion

Here, the effects of two variables, Ins-icv and oral Cinn extract in an AD rat model were investigated. To evaluate the animals’ behavioral performance, the MWM test was applied in two continuous 3-day trials (to find the visible and hidden platform) and a 1-day probe test. The performance of animals in the STZ + Cinn + Ins group was improved in the MWM test observed in our study. Also, the expression of GLUT1, 3, and 4 genes was increased in the hippocampal tissue. Therefore, co-administration of oral Cinn extract and Ins-icv injection increased the sensitivity of cells to Ins and increased the expression of GLUT1, 3, and 4 genes, and its positive results were reflected in the MWM behavioral test.

The MWM is a test that assesses allocentric navigation, a capacity that uses cues outside the organism for navigation (distal cues) and includes the entorhinal cortex, the hippocampus, and surrounding structures. Allocentric navigation is a conserved form of learning and memory for survival, and this converges with semantic and episodic memory systems in humans. Assessing MWM spatial navigation is applied to study the underlying mechanisms of how the brain captures, consolidates, and retrieves allocentric information (20). In our study, the visible platform test was performed before the hidden platform test. Rats are more prone to thigmotaxis, hovering, and other behaviors (such as jumping out of the pool and jumping the rescue platform) that interfere with learning; the visible platform training step not only helps the rat learn but also inhibits unwanted behavior. Previous studies have shown that in hidden platform conditions, rats use the distance to the pool’s edge as a cue to reach the platform in addition to at least two distal cues (21). In general, rats do not move based on absolute position, but relative position relative to directional cues (20).

STZ-icv was applied to induce a model of AD in rats. STZ-icv in rodents was practiced to produce an animal model of dementia. Chronically decreased cerebral glucose uptake and multiple other effects that resemble molecular, pathological, and behavioral features of AD were indicated in single or double STZ-icv injections (22). AD was induced in rats by icv administration of STZ; the model was confirmed by behavioral and molecular results when comparing the STZ group with the negative control. Our results in the STZ group showed that STZ-icv injection disrupted the spatial memory, which led to an increase in the escape latency time in the visible and hidden platform and reduced time spent in the target quadrant in the probe test. The results, however, improved in the STZ+Ins treatment groups. Shingoa *et al*. (2013) showed that one of the side effects of STZ-icv is a decrease in the insulin-degrading enzyme (IDE) and an increase in Aβ deposition in the hippocampus. However, the administration of a long-acting Ins analog into the third ventricle (3V) could change the level of the hippocampus’ IDE (8). Previous research has shown that STZ-icv injection reduces hippocampal choline acetyltransferase (ChAT) activity, due to impaired brain energy metabolism caused by STZ-icv and subsequent damage to cholinergic neurons (10). Increased Ins levels in the brain are believed to cause limited clearance of amyloid peptides because they both compete for the common IDE mechanism (9). STZ-icv injection disrupts the glucose transport system and metabolic pathways under the control of Ins signaling. It also inhibits the synthesis of ATP and acetyl CoA and reduces cholinergic activity and cognitive impairment (8). In the visible and hidden platform behavioral tests, no significant difference was obvious between the Ins control group and STZ+Ins; however, this was not true for the probe test. 

The mRNA expression of GLUT1, GLUT3, and GLUT4 was higher in the Ins control compared with the STZ+Ins treatment group. Specific transporters to cross plasma membranes are provided by glucose, as a major source of brain energy. BBB is equipped with a particular carrier system, which mediates the uptake of D-glucose across BBB and the delivery of D-glucose to astrocytes and neurons. They regulate the need for energy through glucose in response to various neural activities. To date, 10 GLUT proteins have been recognized in the nervous system. Between them, a pivotal role in the glucose uptake of the brain belongs to GLUT1 and GLUT3; GLUT4 is present in some neurons in the brain, hippocampal and cerebellar neurons (6). GLUT1 is an insulin-sensitive glial that takes up glucose from the plasma membrane for neuronal consumption. Thus, GLUTs and astrocytes contribute to the cellular glucose balance of the brain (5). The mRNA levels of GLUT 1, 3, and 4 in the hippocampus in all groups were evaluated. In the STZ group, the expression of all these genes was reduced significantly, whereas, in the Cinn control group, it was increased. It was reported that due to high levels of Aβ in these regions, the under-expression of GLUT1 and GLUT3 mRNA occurred in hippocampal and cortical regions in patients and rodent models of AD. Moreover, the onset of clinical symptoms appears as a result of GLUT3 reductions (23). The insulin-sensitive GLUT4 protein is present in the brain which is overexpressed and translocated in the brain tissues (24). In our study, the under-expression of these genes was significantly obvious in the STZ group. It was reported that both mechanisms including impaired Ins expression and/or signaling mechanisms are involved in AD (25). The activity of the glycogen synthase kinase-3 α/β (GSK-3α/β) is modulated by PI3-K activated protein kinase B (PKB), known as AKT regulates. The hyperphosphorylation of tau protein is contributed by GSK3β (26). Increased cellular glucose uptake is regulated upon activation of PI3-K to stimulate the translocation of GLUT4. Reduction of glucose uptake and utilization of brain tissue occurs during diminishing in GLUT4 translocation due to Ins resistance in AD. The metabolism of the amyloid-β protein precursor (AβPP) and Aβ peptide (Aβ) also takes place following activation or inhibition of the PI3-K pathway. In animal models, increased levels of phosphorylated tau protein in the brain following the loss of insulin-mediated activation of the PI3-K pathway was reported(27). A reduction in the phosphorylation of tau protein which promotes its binding to microtubules was reported subsequent to the Ins stimulation (28) and regulation of soluble APP release (29). A remarkably increased level of Aβ 1-42 and decreased β subunit of the Ins receptor, IGF-1 receptor, IRS-1, IRS-2, as well as the p85 subunit of PI3-K in the brain of animal models of AD was also noticed to have significantly increased by Chua *et al*. (30) . An undetermined Ins signaling and decrease in the translocation of GLUT4 from intracellular compartments to the plasma membrane was induced by Aβ42 oligomers (31). 

The effect of Ins-icv in the AD and non-AD groups was studied to compare the action of Ins in behavioral and molecular tests. In some cases, a statistically significant difference between the Ins control groups compared with the Ins treatment groups was observed. Essential role for Ins in brain glucose and lipid metabolism was provoked by over-expression of Ins receptors in the brain. However, A smaller amount of Ins is also formed in pyramidal neurons in the hippocampus, prefrontal cortex, entorhinal cortex, and the olfactory bulb (32). Circulating Ins through active receptor-mediated transmission can cross the BBB and affect its receptors in the brain. These receptors are not evenly distributed in the brain but the hippocampus is a pivotal site. Ins receptors and signaling can affect various mechanisms such as the buildup of neurotransmitters, calcium influx, synaptic connections, apoptosis, and neurogenesis (1). The shortage in Ins signaling results from the effects of Ins resistance and deficiency (33); thus, the concept of AD was suggested as “type 3 diabetes” (34). Since impaired hippocampal Ins signaling leads to memory impairment (35), Ins resistance and hyperinsulinemia have a possible link with type 3 diabetes (36).

The effect of oral Cinn on behavioral functions and mRNA expression of GLUT in our animal AD model was also investigated. In this study, oral Cinn was evaluated solely and in combination with Ins in the control and treatment groups. In the behavioral test, the latency time to find the visible platform was demolished in the Ins+Cinn control in comparison with the STZ+Ins group. These results were similar in the probe test, but in the hidden platform test, despite the reduction in latency time in the Ins+Cinn control group, no considerable difference was shown in the treatment groups. Alternatively, in the study of GLUT 1, 3, and 4 gene expressions in control and treatment groups, the groups treated with Ins+ Cinn had more gene expression than the control and insulin-only treatment groups. These results could raise the possibility of the synergistic effect of Ins and Cinn. This effect of Cinn along with Ins can be due to its bioactive compounds. Enhanced expression of proteins that play a critical role during glucose transport, Ins signaling, and the regulation of dyslipidemia, was also investigated due to the bioactive compounds of Cinn oil. Tissue damage occurs following disruption in the balance between free radicals and oxidative stress. Oxidative stress at a high level increases Ins (12). Improved spatial memory and condition avoidance memory after treatment with cinnamic acid, in STZ-injected mice, were reported by other researchers (2018). The outcomes of the MWM test also verified the influence of cinnamic acid on magnifying spatial memory in diabetic and nondiabetic mice. They suggested decreased lipid peroxidation in the brain, an increase in glutathione peroxidase, superoxide dismutase (SOD), and catalase activities in the brain, and decreased ROS and nitrite levels by administering cinnamic acid (15).

*P<0.001 different from the STZ; #*P*<0.001 significant difference from STZ + Ins + Cinn ; † *P*<0.001 different from STZ+Cinn ;††*P*<0.001 different from Cinn; ‡ *P*<0.001 different from Cinn+Ins

STZ: Streptozotocin); Ins: Insulin; Cinn: Cinnamon; icv: intracerebroventricular

**Table 1:** Sequences used in the RT-PCR reaction for the GLUT1, 3, and 4 genes expression for evaluation of insulin and cinnamon in streptozotocin-induced Alzheimer’s model in rats

**source**	**Nucleotide sequences(5´-3´) of the primers**	**primers**	**Gene name**
Flessner *et al*. (2012)	**TCAACACGGCCTTCACTG**	**Forward**	**GLUT 1**
	CACGATGCTCAGATAGGACATC	Reverse	
	**TTCTGGTCGGAATGCTCTTC**	**Forward**	**GLUT 3**
	AATGTCCTCGAAAGTCCTGC	Reverse	
	**GTAACTTCATTGTCGGCATGG**	**Forward**	**GLUT 4**
	AGCTGAGATCTGGTCAAACG	Reverse	
	**CAAGGTCATCCATGACAACTTTG**	**Forward**	**GAPDH**
	GTCCACCACCCTGTTGCTGTAG-	Reverse	

*P<0.001 different from the STZ; †*P*<0.001 different from STZ+Cin; # *P*<0.007 different from STZ+Ins

STZ: Streptozotoci); Ins: Insulin; Cinn: Cinnamon; icv: intracerebroventricular

*P<0.001 different from STZ; #*P*<0.001 different from Ins; †*P*<0.001 different from STZ+Ins+Cinn

*P<0.001 different from the STZ; #*P*<0.001 different from the STZ+Ins+Cinn; †*P*<0.001 different from the Ins group; ‡*P*<0.001 different from Cinn+Ins

## Conclusion

In this study, the effect of Ins-icv and oral Cinn extract was assessed on memory improvement in a rat Alzheimer’s model (induced via STZ-icv). In the STZ+Cinn+Ins group, the performance of animals in the MWM test was improved; also, the expression of GLUT1, GLUT3, and GLUT4 genes in hippocampal tissue was increased. In general, we suggest the positive effect of Cinn and Ins to improve the function of the STV-icv AD model in MWM and increase the expression of GLUT1, GLUT3, and GLUT4 genes in the hippocampal tissues. 

In our study, the expression of GLUT genes and the behavioral results were consistent. However, to clarify whether the results of mRNA levels reflect the behavior of rats, supplementary experiments should be performed at the protein level.

## Authors’ Contributions

ES provided concepts, design, definition of intellectual content, research studies, manuscript preparation, manuscript editing, manuscript review, and data acquisition; JS provided concepts, design, definition of intellectual content, manuscript editing, manuscript review, data acquisition, and support; SH helped with manuscript editing and manuscript review, and gave molecular section advice; MT gave behavioral section advice and helped with manuscript editing and review.

## Funding

The authors received no financial support for the research, authorship, or publication of this article.

## Ethical Consideration

The procedures adopted in this study followed Shiraz University’s ethical guidelines for use of animals in experimental studies. 

## Availability of Data and Material

No additional data are available.

## Conflicts of Interest

The authors whose names are listed above certify that they have no affiliations with or involvement in any organization or entity with any financial interest or non-financial interest in the subject matter or materials discussed in this manuscript.
